# Drug-drug interactions in inpatient and outpatient settings in Iran: a systematic review of the literature

**DOI:** 10.1186/2008-2231-22-52

**Published:** 2014-06-25

**Authors:** Ehsan Nabovati, Hasan Vakili-Arki, Zhila Taherzadeh, Mohammad Reza Hasibian, Ameen Abu-Hanna, Saeid Eslami

**Affiliations:** 1 Student Research Committee, Department of Medical Informatics, Faculty of Medicine, Mashhad University of Medical Sciences, Mashhad, Iran; 2 Department of Health Information Management/Technology, School of Allied Health Professions, Kashan University of Medical Sciences, Kashan, Iran; 3 Neurogenic Inflammation Research Center, Faculty of Medicine, Mashhad University of Medical Sciences, Mashhad, Iran; 4 Targeted Drug Delivery Research Center, School of Pharmacy, Mashhad University of Medical Sciences, Mashhad, Iran; 5 Pharmaceutical Research Center, School of Pharmacy, Mashhad University of Medical Sciences, Mashhad, Iran; 6 Department of Medical Informatics, Faculty of Medicine, Mashhad University of Medical Sciences, Mashhad, Iran; 7 Department of Medical Informatics, Academic Medical Center, University of Amsterdam, Amsterdam, The Netherlands

**Keywords:** Adverse drug events, Developing countries, Drug-drug interaction, Medication errors, Incidence, Intervention, Iran

## Abstract

Drug-drug interactions (DDIs) are an important type of adverse drug events. Yet overall incidence and pattern of DDIs in Iran has not been well documented and little information is available about the strategies that have been used for their prevention. The purpose of this study was to systematically review the literature on the incidence and pattern of DDIs in Iran as well as the used strategies for their prevention. PubMed, Scopus, electronic Persian databases, and Google Scholar were searched to identify published studies on DDIs in Iran. Additionally, the reference lists of all retrieved articles were reviewed to identify additional relevant articles. Eligible studies were those that analyzed original data on the incidence of DDIs in inpatient or outpatient settings in Iran. Articles about one specific DDI and drug interactions with herbs, diseases, and nutrients were excluded. The quality of included studies was assessed using quality assessment criteria. Database searches yielded 1053 potentially eligible citations. After removing duplicates, screening titles and abstracts, and reading full texts, 34 articles were found to be relevant. The quality assessment of the included studies showed a relatively poor quality. In terms of study setting, 18 and 16 studies have been conducted in inpatient and outpatient settings, respectively. All studies focused on potential DDIs while no study assessed actual DDIs. The median incidence of potential DDIs in outpatient settings was 8.5% per prescription while it was 19.2% in inpatient settings. The most indicated factor influencing DDIs incidence was patient age. The most involved drug classes in DDIs were beta blockers, angiotensin-converting-enzyme inhibitors (ACEIs), diuretic agents, and non-steroidal anti-inflammatory drugs (NSAIDs). Thirty-one studies were observational and three were experimental in which the strategies to reduce DDIs were applied. Although almost all studies concluded that the incidence of potential DDIs in Iran in both inpatient and outpatient settings was relatively high, there is still no evidence of the incidence of actual DDIs. More extensive research is needed to identify and minimize factors associated with incidence of DDIs, and to evaluate the effects of preventive interventions especially those that utilize information technology.

## Introduction

Adverse drug events (ADE) are the most common complications related to medication therapy among patients [1-3]. ADEs are common, costly, and may have life-threatening consequences [4-6]. The high incidence of medication use in medical therapy and possibility of human errors increase the incidence risk of these adverse events.

Drug-drug interactions (DDIs) are an important subgroup of ADEs [7] which are highly prevalent in patients receiving multiple-drug treatment [8]. DDIs may lead to severe adverse events which can result in patient hospitalization. Some studies have estimated that up to 3% of hospital admissions are caused by DDIs [9-11].

Although it is widely recognized that DDIs may harm patients, their incidence is still high [12]. The majority of these interactions occurred because either prescribers do not consider them relevant [13] or prescribers’ knowledge of DDIs is generally poor [14]. Hence, they could be prevented through applying proper interventions. This can improve the quality of drug therapy and increase patient safety. Interventions aimed at reducing DDIs are likely to be more effective, if before their development, the incidence and pattern of DDIs are determined accurately.

Estimates about the incidence of DDIs in different countries vary from 6% to 70% due to variability in methodologies and settings [12,15-18]. Because of this variation, it is important that the related evidence is aggregated and summarized in each country, separately. To our knowledge, three systematic reviews in the literature reviewed DDIs studies. Espinosa-Bosch et al. conducted a review on English and Spanish studies which had reported incidence of DDIs in hospital care [19]. They showed that around 20% of hospitalized patients were susceptible to DDIs and incidence was higher in patients with heart disease and the elderly. Another review has summarized and described findings from studies that assessed harmful DDIs in elderly patients [20]. It has been conclusively shown that significant harm is associated with DDIs in elderly patients. Also, Riechelmann and Giglio systematically reviewed the studies, published in English, Portuguese, and Spanish, on the frequency of DDIs in cancer patients [21]. They estimated that about one-third of cancer patients are at the risk of DDIs.

None of the DDIs systematic reviews were conducted in a developing country. In Iran, several DDIs studies have been conducted, but there is uncertainty about their overall incidence, pattern of the most involved medication classes, and the possible interventions and their effectiveness.

The objective of this systematic review is to identify and summarize all evidence concerning DDIs in Iran as an example of a developing country. In this study we address four questions: (1) what is the incidence and pattern of DDIs?; (2) which factors are associated to incidence of DDIs?; (3) what interventions have been used to prevent this type of medication errors?; (4) which interventions have been effective in reducing DDIs?

## Methods

### Search strategy and data sources

A comprehensive search strategy for original articles was developed using terms related to drug interaction (drug interaction, adverse drug event, adverse drug reaction, medication error, prescription error) combined with terms related to Iran (Iran, Iranian).

The following electronic databases were searched for English articles using customized search strategies: MEDLINE/PubMed and Scopus. Persian Electronic databases including Scientific Information Database (SID), IranMedex, IranDoc, and MagIran were searched using Persian terms equivalent to the English terms mentioned above. The electronic databases were last searched on March 2013. To ensure that no article is missed, we also searched Google Scholar using both Persian and English search terms.

In a final search, the reference lists of all identified articles were also reviewed to identify additional relevant articles (snowball method).

### Inclusion and exclusion criteria

All published studies on children, adults, and elderly patients that were conducted in either an outpatient or inpatient setting in Iran and published either in English or Persian were included. Various types of research designs including observational studies that reported the incidence of DDIs and interventional studies that evaluated an intervention on reduction of DDIs were included.

Articles about one specific DDI and drug interactions with herbs, diseases, and nutrients were excluded. Moreover, we excluded letters, opinions, conference papers, and dissertations.

### Review procedure and data extraction

A reviewer conducted the search for the articles. Two reviewers (including the one conducting the literature search) considered the inclusion and exclusion criteria independently and screened the title and abstract of all potential relevant articles. Any discrepancies on the eligibility of the articles were resolved by discussion among the reviewers. After the inclusion process, the full text of eligible articles for the purposes of this review was retrieved. In the case of inaccessibility, the full text was requested from the authors by email. The full text of each eligible article was reviewed and abstracted into a pre-specified form.

The data abstraction form was used to collect information on the following characteristics: objectives, setting, study period, type of study, sampling, data source, DDIs reference, main findings, details of reported DDIs, most frequent DDIs, factors associated with incidence of DDIs, interventions and their outcomes, and other relevant information.

### Quality assessment of the included studies

There is no tool that assesses the quality of DDIs studies. A twelve-item quality assessment tool (Table 1) was developed based on the criteria taken from the tools for assessing the quality of medication error studies [22,23]. Overall quality scores ranged from 0 to 12 (0 to 6 points = poor, 7 to 9 points = moderate, 10 to 12 points = high). Two reviewers independently scored the quality criteria for each included study and a third reviewer resolved any discrepancies.

**Table 1:** The tool used to rate the quality of the included studies

**Quality assessment criteria**	**Score**
1) Aims/objectives of the study clearly stated	1
2) Definition of what constitutes a DDI	1
3) DDI categories specified	1
4) DDI categories defined	1
5) Mention of DDI reference	1
6) Data collection method described clearly	1
7) Setting in which study was conducted described	1
8) Study subjects described	1
9) Sampling and calculation of sample size described (unit of measurement)	1
10) Potential or actual DDIs assessed	1
11) Measures in place to ensure that results are valid	1
12) Limitations of study listed	1
Maximum score	12 points

Each item is related to a quality assessment criterion with score 0 or 1.

Due to variations in the methods used to report on DDI statistics, we mainly reported qualitative aggregate results.

## Results

### Literature search results

**Figure 1:**
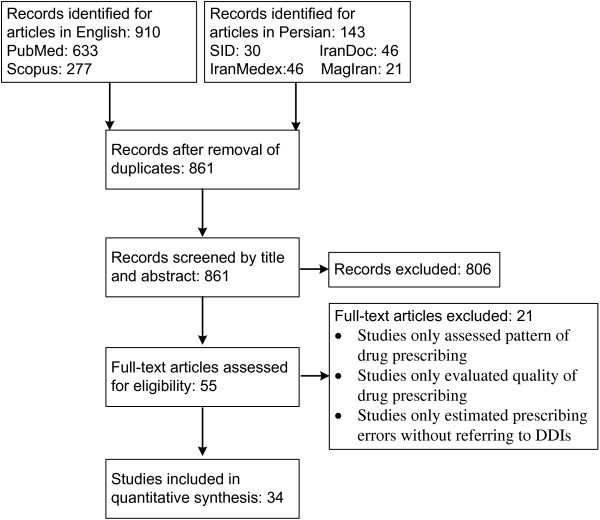
**Flow diagram of the literature search and study selection.** The search strategy focused on studies that analyzed original data on the incidence of DDIs in inpatient or outpatient settings in Iran.

The flow diagram of literature search is shown in Figure 1. Electronic literature search on MEDLINE/PubMed, Scopus and Persian databases identified a total of 1053 records. 861 unique records remained after excluding duplicates. After reviewing titles and abstracts and applying inclusion and exclusion criteria, 54 articles were chosen for full text review. By hand-searching the references list, two additional relevant articles were also identified. Subsequently, the full texts of these potentially relevant articles were obtained except one [24] (even after contacting its authors by email). After detailed full text review of 55 articles, a further 21 articles were excluded, because they only assessed pattern of drug prescribing, only evaluated quality of drug prescribing, or only estimated prescription errors without referring to DDIs. Finally, 34 relevant articles that met our specified criteria were included in this review.

### General characteristics of the included studies

The oldest study was published in 1997 and the most recent one in 2013. Twenty-one studies (62%) had been written in Persian and 13 (38%) were in English. In terms of study setting, 15 (44%) and 19 (56%) studies have been conducted in inpatient and outpatient settings, respectively. In terms of study design, 31 studies (91%) were observational and three (9%) were experimental. The majority of studies, 20 out of 34, had used Drug Interaction Facts as their DDI compendia. Table 2 shows the general characteristics of the included studies.

**Table 2:** General characteristics of the included studies

**Ref**	**Year**	**Language**	**Target Population**	**Setting**	**Pathology/Drug type**	**Design**	**Duration**	**Sample Size/Unit of Analysis**		**Drug Interaction Database**
[24]	1997	Persian	All	Outpatient	All	Observational (Retrospective)	2 months	3117 Prescriptions		-
[25]	1997	Persian	All	Inpatient (Internal, Surgery)	All	Observational (Retrospective)	3 months	1000 Prescriptions		-
[26]	1999	Persian	All	Outpatient	Cardiovascular drugs	Observational (Retrospective)	6 months	1038 Prescriptions		Drug Interaction Facts
[27]	2000	Persian	All	Outpatient	All	Observational (Retrospective)	12 Months	4750 Prescriptions		Drug Interaction Facts
[28]	2001	Persian	All	Outpatient	All	Observational (Retrospective)	3 months	1100 Prescriptions		Drug Interaction Facts
[29]	2000	Persian	All	Outpatient	Anti-Depression drugs	Observational (Retrospective)	6 months	3000 Prescriptions		Drug Interaction Facts
[30]	1999-2001	Persian	All	Outpatient	NSAID	Observational (Retrospective)	36 months	1927 Prescriptions		Hansten Drug Interactions
[31]	2000	Persian	All	Outpatient	All	Observational (Retrospective)	6 months	3000 Prescriptions		Drug Interaction Facts
[32]	2000	Persian	All	Outpatient	All	Observational (Retrospective)	12 months	1800 Prescriptions		Drug Interaction Facts
[33]	2001	Persian	All	Outpatient	All	Observational (Retrospective)	6 months	5300 Prescriptions		Drug Interaction Facts
[34]	2005-2006	Persian	All	Outpatient	All	Interventional (Quasi Experimental)	6 months	5300 Prescriptions – 43 Prescribers		Drug Interaction Facts
[35]	2002-2003	Persian	All	Outpatient	All	Interventional (Quasi Experimental)	12 months	6704 Prescriptions – 119 Prescribers		Drug Interaction Facts
[36]	2006	Persian	All	Inpatient	All	Observational (Retrospective)	1 month	6969 Prescriptions		-
[37]	2004	Persian	Pediatrics	Inpatient	All	Observational (Retrospective)	6 months	898 Medical Records		Drug Interaction Facts
[38]	2008	Persian	All	Outpatient	All	Observational (Retrospective)	6 months	167305 Prescriptions		-
[39]	2005 – 2006	Persian	All	Outpatient	Dental	Observational (Retrospective)	6 months	666 Prescriptions		-
[40]	2009	Persian	war-injured veterans with Psychiatric disorders	Outpatient	All	Observational (Retrospective)	3 months	150 Patients		Food and Drug Administration Package
[41]	2006 – 2007	Persian	All	Inpatient	All	Observational (Retrospective)	6 months	400 Medical Records		Hansten Drug Interactions
[42]	2009 – 2010	Persian	All	Inpatient (ICU)	All	Observational (Retrospective)	12 months	371 Medical Records		Drug Interaction Facts
[43]	2010	Persian	war-injured veterans with Psychiatric disorders	Inpatient	All	Observational (Retrospective)	4 months	1435 Patients		Food and Drug Administration Package
[44]	2009	Persian	Elderly	Inpatient (ICU)	All	Observational (Retrospective)	12 months	70 Patients		Drug Interaction Facts
[45]	2000	English	All	Inpatient (ICU, CCU, internal and infectious)	All	Observational (Retrospective)	6 months	3130 Prescriptions		Drug Interaction Facts
[46]	2002	English	All	Outpatient	All	Interventional (Quasi Experimental)	6 months	5600 Prescriptions		Drug Interaction Facts
[47]	2000	English	Elderly	Outpatient	All	Observational (Retrospective)	2 months	3000 Prescriptions		Drug-Reax (Micromedex)
[48]	2005	English	All	Inpatient (ICU)	All	Observational (Retrospective)	6 months	567 prescriptions		Drug Interaction Facts
[49]	2006 – 2008	English	All	Outpatient	All	Observational (Retrospective)	24 months	11,562,808 prescriptions		Drug Interaction Facts
[50]	2007 – 2009	English	All	Outpatient	All	Observational (Retrospective)	30 months	44,567,750 Prescriptions		Drug Interaction Facts
[51]	2005 – 2006	English	Elderly	Outpatient	All	Observational (Retrospective)	12 months	2041 Patients		Swedish Classification System
[52]	2001	English	All	Inpatient	All	Observational (Prospective)	3 months	519 Prescriptions		Drug Interaction Facts
[53]	2010	English	Adults	Inpatient	All	Observational (Retrospective)	12 months	1000 Prescriptions		A computerized DDI database system (Prescription Analyzer 2000, Sara Rayane Co., Iran)
[54]	2012	English	All	Inpatient (ICU)	All	Observational (Prospective)	20 days	101 patients		Drug Interaction Facts
[55]	2011 – 2012	English	All	Inpatient (hematology-oncology ward)	Cancer Patients/Anti-Cancer and Non-Anti-Cancer drugs	Observational (Prospective)	6 months	83 patients		On-Desktop Lexi-Interact
[56]	2011	English	All	Inpatient (Post-ICU)	All	Observational (Prospective)	6 months	203 patients		Online Lexi-Interact
[57]	2009 – 2010	English	All	Inpatient (hematology-oncology ward)	Cancer Patients	Observational (Retrospective)	12 months	224 patients		Drug Interaction Facts
	**Range: 1997 – 2013**	**English: 13 Persian: 21**	**All Populations: 27**	**Outpatient: 19**		**Observational: 31**	**Minimum: 1 month**	**Prescription: 23**	**Prescriptions:**	**FACT: 20**
									**Minimum: 519**	
									**Maximum: 44,564,650**	
			**Elderly: 3**	**Inpatient: 15**		**Interventional: 3**	**Maximum: 36 months**	**Patient: 11**	**Patients:**	**Others: 9**
			**War-injured: 2**						**Minimum: 70**	
				**ICU: 6**					**Maximum: 2041**	
			**Pediatrics: 1**	**All Dep.: 9**			**Mod: 6 months (38%)**			**Not Stated: 5**
			**Adults: 1**							

### Quality of the included studies

After the quality assessment of individual studies, none of them fulfilled all the quality criteria. Three studies (9%) were of higher quality (10 points), 16 studies (47%) were of moderate quality (7 to 9 points), and 15 studies (44%) were of poor quality (0 to 6 points). In terms of the quality assessment criteria, no study assessed actual DDIs, only four studies (12%) listed their limitations, and 15 studies (44%) defined DDIs categories.

### Findings of the included studies

Twenty-five (73.5%) studies reported the overall incidence of potential DDIs in the study population (prescription or patient). Nine studies (26.5%) have not reported the overall incidence of DDIs. Among the studies performed in outpatient settings, nine studies assessed the overall incidence of potential DDIs in prescriptions in the population for all types of drugs. The median incidence of potential DDIs in prescriptions of these studies was 8.5% (Interquartile Range (IQR): 8.4-10.1). The other outpatient studies focused on the incidence of potential DDIs in cardiovascular drugs (DDIs percentage = 50%) [26], non-steroidal anti-inflammatory drugs (NSAIDs) (DDIs percentage = 49%) [30], antidepressant drugs (DDIs percentage =22%) [29], dental drugs (DDIs percentage =27%) [39], and elderly people (DDIs percentage =10% and 14%) [47,51].

Among the studies performed on inpatient prescriptions, four assessed the overall incidence of potential DDIs in prescriptions for all groups of patients in all departments and for all drug classes [24,25,52,53]. The median incidence of potential DDIs in these studies was 19.2% (IQR: 15.5-22). The focus of one study in inpatient setting was on pediatric patients (DDIs percentage = 21%) [37]. The two studies that focused on potential DDIs in hospitalized patients in the hematology and oncology departments reported the incidence of 38% and 63% [55,57].

More than half of the studies (21 studies, amounting to 62%) have grouped the identified DDIs in terms of severity and reported the percentage of major, moderate, and minor DDIs separately. The median percentage of major, moderate, and minor DDIs in these studies were 7.7% (IQR: 4.4-11.6), 67.4% (IQR: 51.3-75.3), and 24.2% (IQR: 16.4-41.9), respectively. Six additional studies (17.5%) have calculated the percentage of prescriptions with at least one DDI grouped by severity. The median percentage of prescriptions with major, moderate, and minor DDIs were 0.8% (IQR: 0.7-1.3), 10.2% (IQR: 5.6-11.2), and 9.6% (IQR: 3.6-22.8), respectively.

Fifteen studies (44%) have confirmed the association between the number of medications and the incidence of DDIs. The influence of other factors on incidence of DDIs was mentioned in 11 studies (32%). These factors are listed in Table 3.

**Table 3:** Factors associated with incidence of DDIs

**Factors**		**Description**
**Physician**	**Gender**	The DDI incidence was significantly higher among male doctors [33,46].
	**Age**	Older physicians prescribed medications with more major DDIs than younger physicians (Not statistically significant) [46].
	**Specialty**	Major DDIs were higher in the prescriptions of specialist practitioners in comparison to general practitioners (cardiologists and internists ranked top on the list, while dermatologists ranked the lowest) [50].
		General practitioners had more prescriptions with major DDIs than specialists (statistically significant) [33].
		Significant level 1 DDIs^1^ were higher in prescriptions of internal specialists and cardiologists than other practitioners [31].
		Significant level 2 DDIs^1^ were higher in prescriptions of obstetrician and gynecologist than other practitioners [31].
		Significant level 3 DDIs^1^ were higher in prescriptions of general physicians than specialists [31].
		General physicians prescribed more medications with major DDIs than specialists (Not statistically significant) [46].
	**Number of Prescriptions**	Physicians with 150 or more prescriptions in one month had more DDIs than the others (statistically significant) [33].
**Patient**	**Gender**	DDIs were significantly higher in female patients [53].
		DDIs were significantly higher in male patients [42].
	**Age**	Clinically relevant DDIs were more common for patients 75 years or above than other patients [51].
		DDIs were significantly higher in patients aged over 60 years than other patients [42,53,57].
	**Disease**	DDIs were significantly higher in cardiology patients than other patients [53].
		DDIs were higher in Hematologic cancer patients than patients suffer from other diseases [57].
		DDIs were higher in patients whose source of cancer was in different specific organs than other cancer patients [57].
	**Length of Hospital Stay**	DDIs were higher in patients with longer hospital stay than other patients [42,57].
	**Department**	DDIs occurred in surgery department more than the other departments [36].
	**Drug**	DDIs was significantly higher in patients who have been prescribed digoxin than other patients [53].

^1^Significance rating is based on Drug Interactions Facts™. The factors are related to physicians and patients’ characteristics.

Twenty three studies (67.6%) have determined the most frequent DDIs. Among them, eight studies have also classified the most frequent DDIs by severity. The most frequent major DDIs in the studies, which ranked in the first 10 identified DDIs, are listed in Table 4. As this table shows, five studies have ranked the major interaction between digoxin and furosemide among the most frequent interactions.

**Table 4:** The most frequent major DDIs

**The most frequent major DDIs**	**References**
Digoxin + Furosemide	[25-27,50,53]
Captopril + Triamterene-H	[38,50]
Carvedilol + Salbutamol(Albuterol)	[56,57]
Aspirin + Clopidogrel	[56]
Clopidogrel + Omeprazole	[56]
Pantoprazole + Clopidogrel	[56]
Aspirin + Warfarin	[56]
Haloperidol + Propranolol	[50]
Amitriptyline + Clonidine	[50]
Chlorpromazine + Propranolol	[50]
Propranolol + Verapamil	[50]
Amiodaron + Digoxin	[50]
Gemfibrozil + Atorvastatin	[50]
Cyclosporine + Fluconazole	[55]
Cyclosporine + Phenytoin	[55]
Atorvastatin + Fluconazole	[55]
Lovastatin + Gemfibrozil	[55]
Arsenic Trioxide + Fluconazole	[55]
Aspirin + Ibuprofen	[32]
Theophylline + Propranolol	[32]
Pseudoephedrine + Furazolidone	[32]
Dextromethorphan + Furazolidone	[32]
Tranylcypromine + Levodopa	[29]
Clomipramine + Furazolidone	[29]
Clonazepam + Olanzapine	[43]
Digoxin + Verapamil	[46]
Rifampin + Isoniazid	[48]
Verapamil + Erythromycin	[53]

Names and classes of drugs which mostly contributed to DDIs have been reported by 14 studies (Table 5). Beta blockers, angiotensin-converting-enzyme inhibitors (ACEIs), diuretic agents, and NSAIDs have been mentioned most often as drug classes. Digoxin contributed the most to major DDIs.

**Table 5:** The most common drugs contributing to DDIs

**Names and classes of drugs**	**Percentage of identified DDIs**	**Reference**
Beta Blockers	35.21%	[26]
Inotropic Drugs e.g. Digoxin	15.94%	
ACEIs^1^ e.g. Captopril	15.35%	
Diuretics e.g. Furosemide	14.66%	
Calcium Channel Blockers e.g. Diltiazem	7.33%	
Nitrate e.g. Nitrocardin	4.18%	
Antihyperglycemic Drugs e.g. Clofibrate	4.09%	
Antiarrhythmic Drugs e.g. Amiodarone	2.64%	
Digoxin	50% of severe DDIs	[27]
Gentamicin	26.5% of moderate DDIs	
Diphenhydramine Compound	24.85%	[28]
Dextromethorphan-P	15.38%	
Pseudoephedrine	11.8%	
Antibiotics	7.1%	
Tricyclic Antidepressant	72.7%	[29]
MAOIs^4^	25.2%	
SRIs^3^	2.1%	
Antibiotics	Not specified	[24]
Central Nervous System Drugs	Not specified	
NSAIDs^2^	Not specified	
Antidepressant	52%	[40]
Anti Infectives	27.5%	[41]
Other Drugs	21.5%	
Antiarrhythmic	15.5%	
Antihypertensive	11.1%	
Anti-diabetic	5.6%	
Anticoagulant	5.6%	
Diuretics	3.7%	
Hormone	2.5%	
Salicylate	2.3%	
Anticonvulsant	1.8%	
Antidepressant	47.5%	[43]
Belladonna	4.4%	[49]
Phenytoin Compound	4.3%	
Cimetidine	3.8%	
Propranolol Hydrochloride	3.6%	
Gentamicin	3.5%	
Acetylsalicylic Acid	3.5%	
Aluminium MGS	3.4%	
Theophylline	3.3%	
Carbamazepine	2.8%	
Contraceptive LD	2.7%	
Digoxin	Not specified	[50]
Diuretics	Not specified	
HMG CoA Reductase Inhibitors	Not specified	
Allopurinol	Not specified	
ACEIs	Not specified	
Warfarin	Not specified	
Gemfibrozil	Not specified	
Haloperidol	Not specified	
Amiodarone	Not specified	
Clonidine	Not specified	
Cardiovascular Drugs	Not specified	[52]
Digoxin	Most common in severe DDIs	[53]
ACEIs	Most common in severe and moderate DDIs	
Beta Blockers	Most common in moderate DDIs	
Fluoroquinolones	Most common in moderate DDIs	
Antacids	Most common in moderate DDIs	
Phenytoin	Not specified	[54]
Antimycotics for systemic use^5^	31.35%	[55]
Immunosuppressants	13.51%	
Sulfonamides and Trimethoprim	9.73%	
Antiepileptics	8.11%	
Antiemetics and Antinauseants	7.02%	
Antigout Preparations	4.05%	
Corticosteroids for Systemic Use, Plain	3.78%	
Other Antineoplastic Agents	2.43%	
Direct Acting Antivirals	2.43%	
Other Beta-lactam Antibacterials	2.16%	

Names and classes of drugs which mostly contributed to DDIs and percentage of identified DDIs by the relevant studies are shown.

^1^ACEIs: Angiotensin-Converting-Enzyme inhibitors.

^2^NSAIDs: Non-Steroidal Anti-Inflammatory Drugs.

^3^SSRIs: Selective Serotonin Reuptake Inhibitors.

^4^MAOIs: Monoamine Oxidase Inhibitors.

^5^Medication classes categorized by the Anatomical Therapeutic Chemical (ATC) classification system of the World Health Organization.

### Interventional studies

Among the included studies, only three were interventional. All three were quasi experimental and have been conducted in outpatient settings. In the first study [34], the effects of face to face education, information feedback, and pamphlets designation were evaluated. The study shown that potential DDIs in general practitioners and specialists’ prescriptions decreased (severe: 1.6% before vs. 0.24% after, moderate: 10.6% before vs. 2% after, minor: 5.1% before vs. 2.1% after, p-value < 0.001). In the second study [35], individualized feedback and workshop training programs were used. The study mentioned that potential DDIs with first significance degree (based on Drug Interactions Facts™) in general practitioners’ prescriptions reduced significantly (0.4% before vs. 0.05% after interventions, p-value < 0.001). The third study [46] evaluated the effect of face-to-face training, audit feedback, and educational notes on the major DDIs in general practitioners and specialists’ prescriptions. It demonstrated that severe DDIs diminished significantly (1.5% before vs. 0.4% after, p-value < 0.05).

## Discussion

This study aimed to provide an overview of the incidence and pattern of DDIs and associated factors in Iran, as an example of a developing country. This is the first review study that summarizes the available evidence of DDIs in Iran.

We identified and described the results of 34 relevant studies addressing the key questions of this review. The overall quality of DDIs studies in Iran was relatively poor, perhaps due to lack of a standard guideline for designing methodology and reporting results of medication error studies. The median incidence of potential DDIs in prescriptions in outpatient settings was 8.5%, while it was 19.2% in inpatient settings. Patient age was the most reported factor influencing the incidence of DDIs. Only three studies were interventional, and all showed significant reduction in potential DDIs.

Our results show that all DDIs studies in Iran assessed potential DDIs, while no study was performed on actual DDIs. Actual DDIs are interactions that actually lead to adverse clinical events in patients. Espinosa-Bosch et al. found a larger number of studies on potential DDIs than on actual DDIs in developed countries (42 vs. 5 studies) [19]. From eight studies included in the review of DDIs in oncology, six assessed potential DDIs while two reported actual DDIs [21]. Our findings in accordance with those from studies in developed countries confirm that there is little evidence of the incidence of actual DDIs in comparison to potential DDIs in the literature. The reason for this may be that identifying actual DDIs is much more complicated than potential DDIs. The majority of the included studies were retrospective which had used computerized programs to review physicians’ orders and prescriptions and to identify potential DDIs. However, to identify actual DDIs, it is required to find the adverse events and confirm that they are a result of simultaneously administering two drugs in the patient regarding his/her condition. The adverse events from DDIs are either not identified or not documented accurately. It should be noted that due to inherent and recall biases and also ethical considerations, the conduction of study designs for assessing actual DDIs may be challenging.

We showed the overall incidence of DDIs in prescriptions in inpatient and outpatient settings reported by Iranian studies (inpatient: median = 8.5%, IQR: 8.4-10.1; outpatient: median = 19.2%, IQR: 15.5-22). The high incidence of DDIs may be associated with high number of drugs per prescription. The mean number of drugs per prescription in Iran is relatively high [58]. This mean number for the outpatient setting was 3.16 and 3.05 in 2010 and 2011, respectively, and 17% and 15% of these prescriptions involved more than four drugs in those years. No similar review aggregated the reported incidence of DDIs in the general population. The other review studies on DDIs have been conducted on either a specific group of patients, e.g. elderly, hospitalized patients, or specific types of drugs e.g. cardiovascular.

The aggregation and comparison of the results of the included studies showed a wide variability of DDIs incidence estimates in the Iranian healthcare community. Relatively few studies which were performed in the general population in developed countries also showed a wide variability of estimates on incidence of DDIs (i.e. 9.8% in Finland [59], 18.5% in Greece [17]). Moreover, a systematic review on incidence of medication errors in Iran showed a wide variability of estimates [60]. Different study methods, various drug interaction databases, diverse study populations, different sample sizes, and some other factors have caused this considerable variability; therefore, direct comparison between the studies is impossible. Maximum incidence of potential DDIs in prescriptions (50%) was reported in a study which assessed DDIs of cardiovascular drugs in outpatient prescriptions [26]. Similarly, the findings obtained in a study from a developed country showed that 80% of elderly hospitalized patients with heart diseases were susceptible to DDIs [61]. The high number of prescribed drugs and also frequent prescribing of some drugs with many possible DDIs may cause the high incidence of DDIs in this group of patients. One included study in our review reported the incidence of potential DDIs among cancer patients as 37.5% [57]. A study conducted in a developed country has shown that 27% of cancer patients were subject to DDIs [62]. Supporting the results of these studies, a review on DDIs among cancer patients reported that approximately one-third of cancer patients are susceptible to DDIs [21]. High growth in the number of new anti-cancer drugs may be one of the main reasons for this.

Incidence of DDIs may be associated with characteristics of patients, prescribers and pharmacists, or some barriers such as insufficient communication between these groups. Good communication between prescribers and pharmacists is crucial to reduce the risks of DDIs [63]. Among studies conducted in Iran, no study has assessed pharmacists’ factors and communication between participants as determinants of DDIs. One review paper specified potential determinants of DDIs associated with pharmacists’ characteristics [64]. In that review, the relationship between pharmacists and prescribers, quality of signals from surveillance programs, pharmacists’ workload, and also availability, quality, and sensitivity of DDIs softwares have been mentioned as the main potential factors that contribute to the occurrence of DDIs. The Iranian studies showed that having heart disease, being old, and receiving digoxin were the main patient factors associated with high incidence of DDIs. Similarly, the findings from another review on the incidence of DDIs in a developed country highlighted these risk factors [19]. Many studies have emphasized that the high incidence of DDIs in the elderly is due to physiological changes related to age, suffering from multiple diseases, and a high rate of medication use. The results reported by Juurlink et al. [7], which show that digoxin toxicity due to DDIs leads to elderly hospitalization, is in line with the results of the Iranian studies. Concerning prescribers’ factors, DDIs were higher in the prescriptions of male prescribers and physicians with greater number of prescriptions in one month. This may be due to the fact that male and busy physicians may less consider the possibility of DDIs during the prescription phase. So far, no study has assessed pharmacological knowledge of prescribers specifically about DDIs.

Drugs most contributing to major DDIs were digoxin, followed by beta blockers, ACEIs, diuretic agents, and NSAIDs. Digoxin, ACEIs, and diuretic agents are frequently prescribed to patients with heart diseases; therefore, this may be one of the reasons why DDIs are highly prevalent in these patients. These results are in the same line as two other reviews which mainly included studies from developed countries [19,20].

The included studies in this review have used various DDIs compendia, mostly (59%) Drug Interaction Facts. Studies have shown that there are discrepancies between DDI compendia [65,66]. In addition, other studies showed that the various performance measures used (such as accuracy, sensitivity, and specificity) of multiple DDI identifying software vary [67-69]. Therefore, one should consider these discrepancies in the resources when comparing the result of the DDI related studies. Clinical relevancy of DDIs is another important issue that should be considered when interpreting DDI related study results, as well as in practice.

Despite the high incidence of DDIs in Iran, only three studies implemented interventions to reduce them [34,35,46]. Two studies evaluated the effects of educational interventions on reduction of DDIs [34,46] and one study evaluated the effect of audit feedback on the quality of prescriptions [35]. The studies showed significant reduction of DDIs after the interventions. In recent years, computerized systems have been involved in medication error reduction strategies and shown to be effective [70]. Computerized physician order entry systems and drug interaction softwares linked to knowledge bases could detect potential DDIs and alert prescribers to prevent serious outcomes. These systems screen the drug list before finalizing an order. In case of a potential medication error, especially a DDI, alerts are displayed and changes in the prescription can be made. Although numerous studies in different countries mentioned the potential improvement of patient safety by computerized systems, there are no studies published on the evaluation of such systems in Iran.

It should be noted that the present review had several limitations. First, although the comprehensive searches were performed, we may have missed some relevant studies. It may be due to limitations of the Persian search engines. To overcome this limitation, we used several search strategies including searching bibliographies of included studies (snowball method). In addition, we searched Google Scholar using both Persian and English search terms. Second, the methodologies of the included studies in our review were heterogeneous. This made it difficult to aggregate their widely varying results. Therefore, no quantitative meta-analysis has been attempted. Third, we did not include results of the unpublished studies (e.g. dissertations and conference papers) in the review. This may affect our estimations. Finally, some of the included studies in our review had small sample sizes (Table 2) that might have led to bias. These may have limited the generalizability of our results.

Due to the lack of studies addressing actual DDIs among Iranian patients, the incidence of adverse events caused by this type of medication errors remains unknown. It is recommended that future DDIs researches investigate the adverse events of DDIs through closely monitoring the patients who are provided with potentially interacting drugs. The prescribers should be aware of the high incidence of DDIs in their prescriptions. They also need to pay attention to patients who are frequently prescribed potentially interacting drugs (e.g. digoxin, beta blockers, NSAIDs, ACEIs, and diuretic agents). In the absence of studies assessing communication among the drug management team (physician, nurse, and pharmacist), it is suggested that future studies delve into aspects of this communication. Better communication between the team members could lead to a safe pharmacotherapy plan and reduce the risks of adverse events caused by DDIs. In recent years, information technology interventions have been employed to improve medication safety and shown to be effective in reducing the number of potential DDIs. We suggest designing and evaluation of such information technology interventions.

## Conclusion

Although there is a large number of studies on the potential DDIs in Iran, there is still no evidence of the incidence of actual DDIs. The included studies in this review had relatively poor quality and were heterogeneous in their methodologies and reporting. However, almost all studies concluded that the incidence of DDIs in both inpatient and outpatient settings is high. Despite this high incidence, there is a limited number of interventional studies aimed at reducing DDIs incidence. Finally, more extensive research is needed to identify and minimize the factors associated with the incidence of DDIs, and to design and evaluate the effects of interventions especially those that utilize information technology to increase awareness about DDIs and decrease their incidence by the drug management team.

## Abbreviations

DDI: Drug-drug interaction; ADE: Adverse drug event; NSAIDs: Non-steroidal anti-inflammatory drugs; ACEIs: Angiotensin-converting-enzyme inhibitors; MAOIs: Monoamine oxidase inhibitors; SSRIs: Selective serotonin reuptake inhibitors; ATC: Anatomical therapeutic chemical classification; IQR: Interquartile range.

## Competing interests

The authors declare that they have no competing interests.

## Authors’ contributions

SE and EN conceived the study idea and design. SE, HV, and EN participated in the literature search, inclusion process, and data abstraction. SE, ZhT, and EN participated in the methodological quality assessment of the included studies and interpretation of data. EN drafted the manuscript. All authors have been involved in critically revising the manuscript. All authors read and approved the final manuscript.
